# Induction chemotherapy with paclitaxel, ifosfamide, and cisplatin followed by concurrent chemoradiotherapy for unresectable locally advanced head and neck cancer

**DOI:** 10.2349/biij.6.3.e23

**Published:** 2010-07-01

**Authors:** I Chitapanarux, E Tharavichitkul, V Lorvidhaya, P Sittitrai, T Pattarasakulchai

**Affiliations:** 1 Division of Therapeutic Radiology and Oncology, Faculty of Medicine, Chiang Mai University, Thailand; 2 Department of Otolaryngology, Faculty of Medicine, Chiang Mai University, Thailand

**Keywords:** induction chemotherapy, concurrent chemoradiotherapy, head and neck cancer, paclitaxel, ifosfamide, cisplatin

## Abstract

**Objective:**

Induction chemotherapy (IC) and concurrent chemoradiotherapy (CCRT) for locally advanced head and neck cancer has been studied in many clinical trials. This study was conducted to determine the response rate of IC with paclitaxel, ifosfamide, and cisplatin followed by CCRT with cisplatin for this group of patients, and the effect of the entire treatment on survival and time to disease progression.

**Methods:**

Thirty patients with advanced and unresectable head and neck cancer were treated with 2 cycles of induction paclitaxel/ ifosfamide/ cisplatin. If the primary tumor had a complete or partial response, patients were treated with 2 more cycles of IC followed by radiotherapy 70 Gy plus 3 cycles of cisplatin. For those with less than partial response or disease progression were treated according to the discretion of the physicians.

**Results:**

Ninety percent of patients had stage IV disease and 40% of them had primary tumor at maxillary sinus and nasal cavity. One patient (3%) achieved complete response (CR) and 18 patients had partial responses (PR) to IC. CCRT enhanced the response rate, resulting in a total of 3 CR (10%) and 16 PR (53%) to treatment. The median time to progression was 11.5 months. The median overall survival was 27 months. The most severe hematologic toxicity occurred during IC was grade3-4 neutropenia (40%). Grade 3-4 mucositis occurred in 68% of patients during CCRT.

**Conclusion:**

This novel combined-modality treatment program, is toxic but feasible, and can be administered for selected patients with advanced and unresectable head and neck cancer. © 2010 Biomedical Imaging and Intervention Journal. All rights reserved.

## INTRODUCTION

It is generally accepted that the treatment of locally advanced squamous cell carcinoma of head and neck cancer (SCCHN) should involve a combined modality approach. Surgery and/or radiotherapy is the main definitive of therapy in patients with locally advanced SCCHN. Induction chemotherapy followed by concurrent chemoradiotherapy is a promising treatment in this group of patients.

Up to 30-50% of chemonaive locally advanced SCCHN patients treated with cisplatin-5FU (PF) have been reported to achieve a complete response rate at primary sites, with an overall response rate of 70-88%.[1-3] The main toxicities associated with PF-induction therapy are hematological, digestive and mucositis with the majority of events being grade 1 or 2.[2,4] Despite the high overall response rates, the low CR rates at primary sites and the high rate of locoregional recurrence in patients with extensive lymph node disease have been disappointing.[2,4,5]

The results of many phase III trials [6-9] show that adding taxane (T) to the standard cisplatin and 5-FU regimen improved the overall survival rate and led to better organ preservation over a PF regimen. A phase II study combining paclitaxel, ifosfamide and cisplatin (TIP) to treat recurrent and metastatic SCCHN produced a high overall response rates (54%) including prolonged duration of CR and encouraging one-and two-year median survival rates.[10]

As an induction chemotherapy for locally advanced head and neck cancer, a phase II study combining paclitaxel, ifosfamide and carboplatin resulted in a high overall response rate of approximately 80%. [11] Based on the demonstrated activity of this triplet regimen in SCCHN and their non-overlapping toxicity profiles, the combination was promising. This single-center, open-label, non-randomized phase II study evaluated tumor response of induction chemotherapy with paclitaxel, ifosfamide, and cisplatin followed by concurrent chemoradiotherapy with cisplatin for locally advanced head and neck cancer, as well as the therapeutic efficacy on overall survival and time to disease progression.

## PATIENTS AND METHODS

### Patient characteristics

Patients were eligible if they had histologically confirmed SCCHN, at least one bidimensionally measurable lesion, aged 18-70 years, stage III or IV (AJCC staging 2002) disease without distant metastases, an ECOG performance status of 0 to1, and adequate bone marrow/hepatic/and renal function. Patients with primary sites in the oral cavity, oropharynx, larynx, hypopharynx, nasal cavity and paranasal sinus were eligible. Patients were excluded from the study if they had primary sites of the nasopharynx or salivary glands. Patients who had been treated for a previous SCCHN, had severe intercurrent medical illness, or known peripheral neuropathy were also excluded from the study. Patients were required to give written informed consent before inclusion in the study. The institutional review board of the Faculty of Medicine, Chiang Mai University, approved the study. The study was conducted in accordance with the principles of the Declaration of Helsinki.

### Treatment

#### Induction chemotherapy (IC)

Eligible patients underwent 2 cycles of IC (paclitaxel 175 mg/m^2^ in a three-hour infusion on day 1, ifosfamide 1000 mg/m^2^ in a two-hour infusion on day 1-3 with uromitexan 200 mg/m^2^ IV in 15 minutes at the time of administration of ifosfamide, and then at 4 and 8 hours, and cisplatin 60 mg/m^2^ as a 90-minute infusion on day 1). Head and neck examination and repeated CT scan of the head and neck were performed to evaluate the initial response after completion of 2 cycles of IC regimen. If the patients achieved a partial response (PR), or complete response (CR), they received 2 more cycles of the same IC. Patients who achieved stable disease (SD) or progressed from the initial 2 cycles of chemotherapy did not receive the same induction chemotherapy regimen and were treated according to the discretion of the physicians.

#### Concurrent chemoradiotherapy (CCRT)

**Figure 1:**
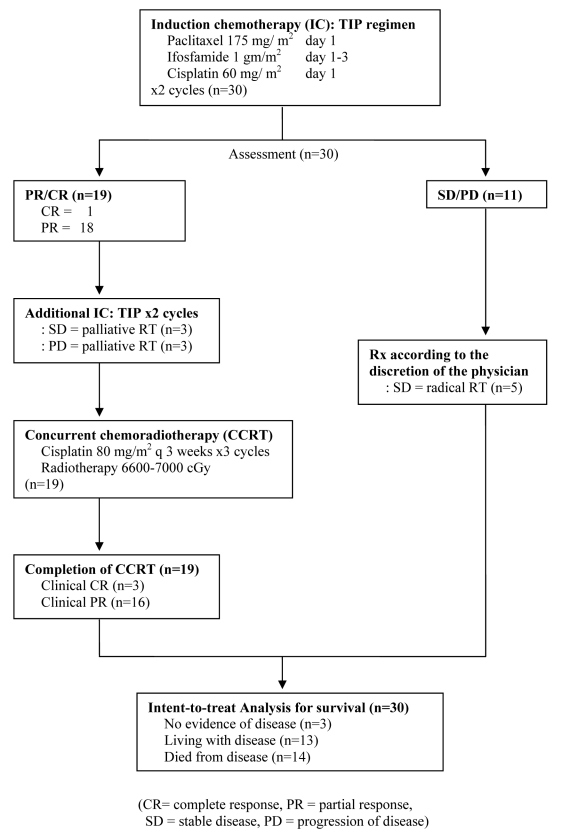
Protocol Schema

Patients who achieved PR or CR after 2 cycles of IC and received additional 2 cycles of IC underwent concurrent with cisplatin 80 mg/m^2^ every 3 weeks for 3 cycles. Radiotherapy was administered once daily as 200 cGy per fraction to a total dose of 6600-7000 cGy. Treatment protocol is shown in Figure 1. During treatment, patients were evaluated for toxicity according to the NCI/CTC version 2.0.

#### Dose Modification

Dose modifications during IC were based on complete blood count (CBC). For ANC <1,500/mm^3^ or platelet <100,000/mm^3^, chemotherapy was delayed for 1 week until recovery and administered with the same dose. If ANC <1,000/mm^3^ or platelet <75,000/mm^3^, chemotherapy was delayed for 1 week until recovery and administered with a reduced dose of 50%. During CCRT; for ANC< 1,500 or platelet < 100,000/mm^3^ or in field toxicity of grade 3, radiotherapy and chemotherapy were withheld until recovery from radiation-induced toxicity of grade <2 and normal CBC.

### Statistical Analysis

Response evaluation was performed after 2 cycles of IC, and 3 months after completion of CCRT in all treated patients. Intent-to-treat analysis was performed. Survival was measured from the date of study entry until date of last follow-up or death. Time to progression was measured as time from the first day of treatment until disease progression.

## RESULTS

Between June 2003 and June 2005, 30 patients with locally advanced SCCHN were accrued to the study. The median age of patients was 53 years, range 30-70 years. All patients had stage III or IV disease and 90% had stage IV disease. The baseline patient characteristics are summarized in Table 1.

**Table 1:** Patient Characteristics

**Characteristics**	**No (%)**
No. of patients	30
Age, median (range)	53 (30-70)
Sex	
Male	21 (70)
Female	9 (30)
ECOG performance status	
0	18 (60)
1	12 (40)
Primary site	
Oral cavity	6 (20)
Oropharynx	8 (27)
Larynx	1 (3)
Hypopharynx	3 (10)
Paranasal sinus + nasal cavity	12 (40)
Stage	
III	3 (10)
IV	27 (90)

### Response to induction chemotherapy

After 2 cycles of IC, all patients were evaluated for response. One of 30 patients had a complete response, 18 patients had partial response, 8 patients had stable disease, and 3 patients had progressive disease. The overall response rate after 2 cycles of IC was 63% (Table 2). Three patients with progressive disease associated with poor performance status were treated with palliative radiotherapy. Among 8 patients who had stable disease, 5 of them received radical radiotherapy and the other 3 patients received palliative radiotherapy due to poor performance status. Only 19 patients who had CR and PR continued their third and forth cycles of IC. The overall response rate at 4 cycles of IC in 19 cases was 100% (Table 2).

**Table 2:** Response Rate

**Response**	**After IC 2 cycles (%)(n=30)**	**After IC 4 cycles (%)(n= 19)**	**After CCRT (%)(n=19)**
CR	1 (3)	1 (5)	3 (16)
PR	18 (60)	18 (95)	14 (74)
SD	8 (27)	-	2 (10)
PD	3 (10)	-	-

### Response after concurrent chemoradiotherapy

Nineteen patients who completed their 4 cycles of induction chemotherapy were treated with concurrent cisplatin and radiotherapy. All of them completed the whole treatment planned. There were 2 additional patients who achieved a complete clinical response at the tumor. Sixteen patients had residual disease after chemoradiotherapy (Table 2). Ten patients did not have salvage surgery because they either had developed distant metastases (2 patients), refused surgery (3 patients), and still had unresectable disease (5 patients). Six patients had salvage surgery following concurrent chemoradiotherapy, five of them eventually developed a neck recurrence, another patient had progression of disease at the primary site.

### Toxicity of induction chemotherapy

Toxicity was assessed in all 30 patients (Table 3). Myelosuppression was frequent, with grade 3-4 neutropenia in 40% of patients. No treatment - related deaths occurred in this study. Neuropathy of any grade was found in 50% of patients. Neurosensory deficits on both hands were common.

**Table 3:** Toxicities (NCI/CTC v.2.0)

**Grade**	**0**	**1**	**2**	**3**	**4**
**Toxicities of IC (% of patients) (n= 30)**					
Anemia	33	60	3	4	-
Neutropenia	-	27	33	37	3
Thrombocytopenia	63	30	-	6	1
Neuropathy	50	50	-	-	-
Fatigue	-	67	33	-	-
Nausea	-	33	67	-	-
Alopecia	-	-	100	-	-
**Toxicities of CCRT (% of patients) (n=19)**					
Anemia	11	63	26	-	-
Neutropenia	16	53	14	16	1
Thrombocytopenia	53	42	-	5	-
Fatigue	-	100	-	-	-
Nausea	-	79	21	-	-
Vomiting	-	74	26	-	-
Radiation induced mucositis	-	5	27	68	-
Radiation induced pharyngitis	-	16	84	-	-
Radiation induced dermatitis	-	74	26	-	-

### Toxicity of concurrent chemotherapy

Toxicity was assessed in 19 patients who received concurrent chemoradiotherapy (Table 3). Seventeen percent of patient had grade 3-4 neutropenia. Severe grade 3 mucositis occurred in 68% of patients. However, prolongation of the RT schedule to 8 weeks was observed in only 2 of 19 patients.

### Time to progression of disease and survival

**Figure 2:**
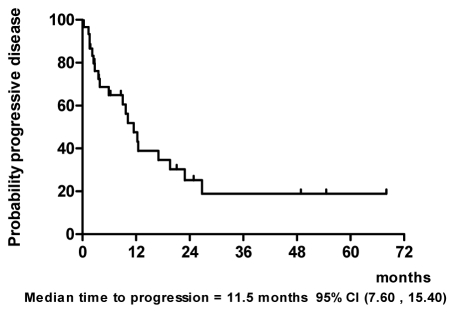
Time to progression.

**Figure 3:**
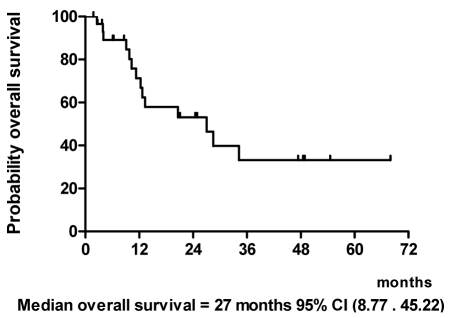
Overall survival.

With a median follow-up of 20.6 months and a range of 6.1 to 68 months as of March 2009, we performed the intent-to-treat analysis in all 30 patients (Figure 1). At the time of analysis, 3 patients (10%) were alive and had no evidence of disease with a minimum follow-up of 39.5 months. Thirteen patients (43%) were alive with disease. Fourteen patients (47%) died from disease. The median time to progression was 11.5 months (Figure 2) and median survival time was 27 months (Figure 3).

## DISCUSSION

Nineteen of 30 patients with locally advanced head and neck cancer who achieved CR or PR after induction chemotherapy of paclitaxel/ifosfamide/cisplatin followed by concurrent radiotherapy with cisplatin, demonstrated overall response rate of 90% and the median survival time of 27 months. Volkes et al. study, focusing on induction chemotherapy with weekly carboplatin and paclitaxel before intensive concomitant chemoradiotherapy with hydroxyurea [12] reported the overall best response of 87%. Our phase II study included patients with stage III/IV disease with 90% of them having stage IV disease which is comparable with their study (96% stage IV). Not surprisingly, our results had a lower survival outcome than other induction chemotherapy studies. This is because, in this study, 11 patients (37%) who had stable disease and progression of disease did not receive further 2 cycles of induction chemotherapy, none of them received concurrent chemoradiotherapy, and six of them (55%) received only additional palliative radiotherapy due to their poor performance status.

Compared to the previous study of Shin et al [11], which had almost the same treatment scheme as ours, except for the regimen of induction chemotherapy, we replaced cisplatin instead of carboplatin, and show a comparable response rate but a much lower survival rate. Unlike our study, Shin et al. found that only 10 of 44 (23%) of patients had stable disease and progressive disease after 2 cycles of induction chemotherapy. Patients then received further treatment with only local therapy, and 4 of 10 (40%) still had no evidence of disease at the time of their analysis. One factor that contributed to the poorer outcome than prior induction studies was the tumor subsite. In our study, the primary site of the paranasal sinus and nasal cavity were much more prevalent than in the other studies (40% versus 0-1%). We knew that the nature of this disease was disappointing, especially in advanced stages, and all 40% of our patients with paranasal sinus cancer were in stage IV of the disease.

There were no therapy-related deaths. Administration of induction TIP regimen was associated with grade 4 neutropenia and thrombocytopenia in only 3% and 1%, respectively. The administration of subsequent concurrent chemoradiotherapy was not compromised. We found only a 1% instance of grade 4 neutropenia during CCRT. Radiation induced mucositis and pharyngitis were common side effects but no one delayed the schedule of radiotherapy.

In summary, we observed a 63% overall response rate of two cycles of induction chemotherapy and as high as 90% after CCRT, and the 27-month median survival with TIP regimen, establishing it as an effective regimen with high but manageable toxicities for locally advanced head and neck cancer. However, the inferior survival outcome to prior studies emphasizes the importance of patient selection according to performance status and primary tumor site to achieve a favorable outcome. Aggressive nutritional support should be considered in patients receiving this regimen, to improve acute palliation and to maximize the delivery of combined-modality therapy. Patients with poor performance status or primary tumors involving the paranasal sinus/nasal cavity may not be good candidates.

## References

[1] Wolf GT (1991). Induction chemotherapy plus radiation compared with surgery plus radiation in patients with advanced laryngeal cancer. The Department of Veterans Affairs Laryngeal Cancer Study Group. N Engl J Med..

[2] Paccagnella A, Orlando A, Marchiori C (1994). Phase III trial of initial chemotherapy in stage III or IV head and neck cancers: a study by the Gruppo di Studio sui Tumori della Testa e del Collo. J Natl Cancer Inst..

[3] Lefebvre JL, Chevalier D, Luboinski B (1996). Larynx preservation in pyriform sinus cancer: preliminary results of a European Organization for Research and Treatment of Cancer phase III trial. EORTC Head and Neck Cancer Cooperative Group. J Natl Cancer Inst..

[4] Domenge C, Hill C, Lefebvre JL (2000). Randomized trial of neoadjuvant chemotherapy in oropharyngeal carcinoma. French Groupe d'Etude des Tumeurs de la Tete et du Cou (GETTEC). Br J Cancer..

[5] Pignon JP, Bourhis J, Domenge C (2000). Chemotherapy added to locoregional treatment for head and neck squamous-cell carcinoma: three meta-analyses of updated individual data. MACH-NC Collaborative Group. Meta-Analysis of Chemotherapy on Head and Neck Cancer. Lancet..

[6] Calais G, Pointreau Y, Alfonsi M (2006). Randomized phase III trial comparing induction chemotherapy using cisplatin fluorouracil with or without docetaxel for organ preservation in hypopharynx and larynx cancer. Proc Am Soc Clin Oncol..

[7] Hitt R, Lopez-Pousa A, Martinez-Trufero J (2005). Phase III study comparing cisplatin plus fluorouracil to paclitaxel, cisplatin, and fluorouracil induction chemotherapy followed by chemoradiotherapy in locally advanced head and neck cancer. J Clin Oncol..

[8] Remenar E, Van Herpen C, Germa Lluch J (2006). A randomized phase III multicenter trial of neoadjuvant docetaxel plus cisplatin and 5-fluorouracil (TPF) versus neoadjuvant PF in patients with locally advanced unresectable squamous cell carcinoma of the head and neck: final analysis of EORTC 24971. Proc Am Soc Clin Oncol..

[9] Posner MR, Haddad RI, Wirth L (2004). Induction chemotherapy in locally advanced squamous cell cancer of the head and neck: evolution of the sequential treatment approach. Semin Oncol..

[10] Shin DM, Glisson BS, Khuri FR (1998). Phase II trial of paclitaxel, ifosfamide, and cisplatin in patients with recurrent head and neck squamous cell carcinoma. J Clin Oncol..

[11] Shin DM, Glisson BS, Khuri FR (2002). Phase II study of induction chemotherapy with paclitaxel, ifosfamide, and carboplatin (TIC) for patients with locally advanced squamous cell carcinoma of the head and neck. Cancer..

[12] Vokes EE, Stenson K, Rosen FR (2003). Weekly carboplatin and paclitaxel followed by concomitant paclitaxel, fluorouracil, and hydroxyurea chemoradiotherapy: curative and organ-preserving therapy for advanced head and neck cancer. J Clin Oncol..

